# Endoscopic ultrasound-guided botulinum toxin injection to prevent mis-injection: improving safety and potential efficacy in spastic esophageal disorders

**DOI:** 10.1055/a-2830-4246

**Published:** 2026-03-24

**Authors:** Abdeldjalil Sais, Jérôme Rivory, Alexandru Lupu, Jean Grimaldi, Mathieu Pioche

**Affiliations:** 1 639305 Department of Gastroenterology and Endoscopy, Groupement Hospitalier Portes de Provence (GHPP), Montélimar, France; 2 Department of Gastroenterology and Endoscopy, Hôpital Edouard Herriot, Hospices Civils de Lyon, Lyon, France

A 69-year-old man presented with disabling solid dysphagia evolving into painful crises despite medical therapy. Initial endoscopy showed no stricture and mild cardiospasm; no mechanical obstruction was observed. A barium esophagogram demonstrated a reduced expansion in the distal thoracic esophagus without stasis and without radiological evidence of lower esophageal sphincter hypertonia.

High-resolution manometry was initially inconclusive, but repeated manometry with a solid provocation reproduced the patient’s “blockage” episodes and showed esophageal shortening with pressurization of the upper half of the esophagus and a few distal spastic contractions, while integrated relaxation pressure remained normal.

Botulinum toxin injection is sometimes used as a rescue therapy in refractory spastic esophageal disorders 1 when per oral endoscopic myotomy is not indicated and was proposed by the motility multidisciplinary team meeting of our hospital. Nevertheless, our experience includes one case of fatal mediastinitis attributable to a misdirected injection of the toxin 2 and a precise protocol of previous antibiotics and directed injection in the muscle layer was proposed following this severe issue.

**Fig. 1:**
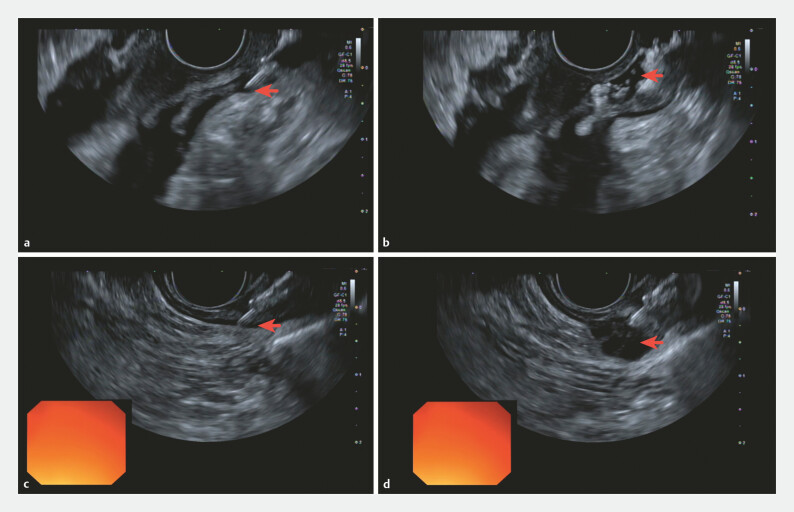
EUS-guided targeting and real-time needle repositioning for botulinum toxin injection. **a** Initial needle insertion into the submucosal layer. **b** Identification of a misdirected injection site located extramurally, posterior to the muscularis propria. **c** Real-time ultrasound-guided repositioning of the sclerotherapy needle into the muscularis propria. **d** Successful intramuscular deposition of the botulinum toxin. EUS, endoscopic ultrasound.

Therefore, we performed injection under linear endoscopic ultrasound (EUS) guidance to maximize intramuscular delivery and minimize extramural mis-injection 3 . Under general anesthesia with intubation, linear EUS confirmed muscular thickening without a clearly predominant segment. A standard endoscopic sclerotherapy needle was used to deliver the botulinum toxin in multiple small volumes along the distal 10 cm of the esophagus ( Video 1 ). Continuous EUS monitoring was used to keep the needle tip within the muscularis propria and avoid injection “behind” the muscle. As shown in the second part of the video, when the needle position was not optimal ( Fig. 1 **a** ), the injection was immediately stopped ( Fig. 1 **b** ), and the needle was repositioned before continuing ( Fig. 1 **c** ), aiming to both prevent adverse events and improve efficacy by ensuring true intramuscular deposition ( Fig. 1 **d** ).

Endoscopic ultrasound-guided botulinum toxin injection to prevent mis-injection in spastic esophageal disorders.Video 1

No immediate adverse event occurred. The patient was discharged with 3 days of amoxicillin–clavulanate. At a 2-month follow-up, the patient reported complete resolution of painful dysphagia crises with significant clinical improvement. EUS-guided targeting may represent a safer and potentially more efficient approach by preventing mis-injection and reducing the risk of rare but serious complications.

Endoscopy_UCTN_Code_CCL_1AB_2AC_3AH Endoscopy_UCTN_Code_TTT_1AO_2AP
